# The Genus *Iodidimonas*: From Its Discovery to Potential Applications

**DOI:** 10.3390/microorganisms10081661

**Published:** 2022-08-17

**Authors:** Seigo Amachi, Takao Iino

**Affiliations:** 1 Graduate School of Horticulture, Chiba University, 648 Matsudo, Matsudo-City 271-8510, Chiba, Japan; 2 Japan Collection of Microorganisms, RIKEN BioResource Research Center, 3-1-1 Koyadai, Tsukuba 305-0074, Ibaraki, Japan

**Keywords:** *Iodidimonas*, iodide oxidation, natural gas brine, multicopper oxidase, disinfection, decolorization

## Abstract

The genus *Iodidimonas* was recently proposed in the class Alphaproteobacteria. *Iodidimonas* strains are aerobic, mesophilic, neutrophilic, moderately halophilic, and chemo-organotrophic. They were first discovered in natural gas brine water containing a very high level of iodide (I^−^). They exhibited a unique phenotypic feature of iodide oxidation to form molecular iodine (I_2_). *Iodidimonas* was also enriched and isolated from surface seawater supplemented with iodide, and it is clearer now that their common habitats are those enriched with iodide. In such environments, *Iodidimonas* species seem to attack microbial competitors with the toxic form I_2_ to occupy their ecological niche. The iodide-oxidizing enzyme (IOX) purified from the *Iodidimonas* sp. strain Q-1 exhibited high catalytic efficiency for iodide and consisted of at least two proteins IoxA and IoxC. IoxA is a putative multicopper oxidase with four conserved copper-binding regions but is phylogenetically distinct from other bacterial multicopper oxidases. The IOX/iodide system could be used as a novel enzyme-based antimicrobial system which can efficiently kill *Bacillus* spores. Furthermore, the IOX/iodide system can be applied to the decolorization of recalcitrant dyes, where iodide may function as a novel inorganic natural redox mediator.

## 1. Introduction

Iodine is an essential trace element in humans and animals because of its important role as a constituent of thyroid hormones. Insufficient dietary iodine can cause iodine deficiency disorders such as endemic goiter and congenital hypothyroidism [1,2]. From a radioecological perspective, the long-lived radioisotope iodine-129 (^129^I, half-life: 1.6 × 10^7^ years) is of concern as it is one of the most persistent radionuclides released from nuclear facilities into the environment [3,4,5,6]. Due to its long half-life, ^129^I is expected to behave similarly to stable iodine (^127^I) over long periods and ultimately, accumulate in the human thyroid glands [7,8]. Therefore, it is necessary to obtain better information on the behavior of iodine in the environment for an accurate safety assessment of ^129^I.

Iodine is primarily present in the ocean, and the predominant chemical forms of iodine in seawater are iodide (I^−^) and iodate (IO_3_^−^), with a total concentration of 0.45 µM [9]. Thermodynamic calculations suggest that the ratio between iodate and iodide (IO_3_^−^/I^−^) in oxygenated seawater should be 10^13.5^, indicating that iodate is the more stable form and that iodide should not be detected in seawater [10]. However, significant quantities of iodide were observed in certain surface waters [11,12,13]. This apparent disequilibrium is considered to be due to the biological reduction in iodate to iodide [14,15,16,17]. To maintain the sustainable cycling of iodine in the ocean, the oxidation of iodide must occur in seawater. However, a kinetic barrier prevents the direct oxidation of iodide to iodate, and this barrier is especially high in the oxidation of iodide to I_2_ [9].

4I^−^ + O_2_ + 4H^+^ → 2I_2_ + 2H_2_O (1)

However, once I_2_ is formed, following its hydrolysis to hypoiodous acid (HIO) and iodide, the disproportionation reaction of HIO to form iodate and iodide may spontaneously occur [9].

I_2_ + H_2_O → HIO + I^−^ + H^+^ (2)

3HIO → IO_3_^−^ + 2I^−^ + 3H^+^ (3)

In 1968, Gozlan [18] reported that the sudden death of fish (*Mugil* sp.) in experimental seawater aquaria was linked to a strong iodine odor and a brownish color. An iodide-oxidizing bacterium was isolated from the aquaria and was later named “*Pseudomonas iodooxidans* sp. nov.” by Gozlan and Margalith [19]. According to their observations, this heterotrophic Gram-negative bacterium oxidizes iodide to I_2_ using an extracellular peroxidase-like enzyme with hydrogen peroxide (H_2_O_2_) as an electron acceptor [19,20].

2I^−^ + H_2_O_2_ + 2H^+^ → I_2_ + 2H_2_O (4)

However, “*P. iodooxidans*” has not been deposited in any culture collection and is not available for verifying the experiments performed by Gozlan and Margalith.

## 2. Discovery of *Iodidimonas* from Natural Gas Brine Waters

**Figure 1:**
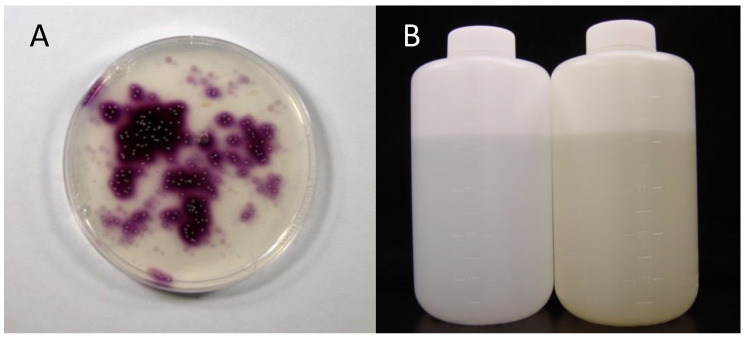
(**A**) Isolation of *Iodidimonas* strains from natural gas brine water. Brine water was spread on the marine agar 2216 medium containing iodide and soluble starch. Note that bacteria of the genus *Iodidimonas* produce purple pigments due to the iodine–starch reaction. (**B**) Enrichment of *Iodidimonas* species in natural seawater. Natural seawater (left) was supplemented with iodide and incubated for 21 days at 30 °C (right). Note that the color of seawater changes to yellow due to the formation of molecular iodine.

Amachi et al. [21] attempted to isolate the “*P. iodooxidans*”-like bacteria from seawater. For this purpose, Marine agar 2216 supplemented with iodide and soluble starch was prepared. If bacteria oxidize iodide to I_2_, colonies are expected to appear purple due to the classical iodine–starch reaction. However, no purple bacterial colonies were obtained from marine samples, including surface seawater, marine sediments, and macroalgae. Just before quitting the screening of iodide-oxidizing bacteria, a unique environmental sample was spread on the medium. Unexpectedly, a substantial number of purple colonies appeared on the medium (Figure 1A). The sample was brine water associated with natural gas (methane) collected from the Boso Peninsula, Chiba, Japan. Brines in this area often have iodine concentrations of approximately 1 mM, which is 2000 times greater than that of natural seawater [22]. The dominant chemical form of iodine in brine is iodide. The production of iodine in this area accounts for 8000 tons annually, which constitutes approximately 25% of the iodine production of the world. Finally, 44 brines collected from various areas in Japan (Chiba, Akita, Niigata, and Miyazaki), the United States, and New Zealand were tested [21]. Of these, 28 showed positive results. The population sizes of iodide-oxidizing bacteria varied from 1.1 × 10^2^ to 8.0 × 10^4^ CFU mL^−1^. Phylogenetic analysis of these bacteria revealed that they were divided into two distinct groups within the class Alphaproteobacteria. One group was most closely related to the aerobic bacteriochlorophyll *a*-producing bacteria *Roseovarius tolerans* and *Roseovarius mucosus,* with sequence similarities of 94–98%. This group is representative of the marine *Roseobacter* group within the family *Rhodobacteraceae*. The other group was most closely related to the halophilic photosynthetic bacterium *Rhodothalassium salexigens,* but the sequence similarity was only 89–91%. This group was later proposed as *Iodidimonas* gen. nov. by Iino et al. [23] (see Section 4).

## 3. Enrichment of *Iodidimonas* in Seawater

To determine whether iodide-oxidizing bacteria inhabit not only natural gas brine water but also natural seawater, Amachi et al. [21] enriched these bacteria by adding 1 mM iodide to the surface seawater collected from various regions in Japan. After incubation for half a year, the color of many seawater samples changed to yellow, suggesting the formation of I_2_ (Figure 1B). As expected, purple bacterial colonies were obtained on the solid medium and were successfully isolated as pure cultures. Interestingly, bacteria isolated from enriched seawater were phylogenetically identical to those isolated from the natural gas brine, including *Roseovarius* and *Iodidimonas* strains. Thus, it was considered that *Iodidimonas* species are widely distributed in surface seawater.

To understand the growth characteristics of the iodide-oxidizing bacteria in an iodide-enriched environment, Arakawa et al. [24] prepared microcosms comprising natural seawater and 1 mM iodide and monitored the transition of microbial communities using a culture-independent technique (PCR-DGGE). The results indicated that bacteria closely related to the genera *Roseovarius* and *Iodidimonas* were predominant in the microcosms after several weeks of incubation. Quantitative PCR analysis revealed that the relative abundance of the genera *Roseovarius* and *Iodidimonas* in the microcosms was 6–75% of the total bacterial population, whereas in natural seawater, it was less than 1%. Furthermore, it was found that *Iodidimonas* sp. strain Mie-8 was more resistant to I_2_ than other heterotrophic bacteria in seawater. These results suggest that I_2_, but not iodide, plays a key role in the growth stimulation of iodide-oxidizing bacteria in iodide-enriched seawater. These bacteria potentially attack competitors using toxic I_2_ to occupy the ecological niche in these environments. As I_2_ is a known strong oxidizing agent and corrodes iron, *Roseovarius* and *Iodidimonas* species sometimes cause the corrosion of carbon steel pipes [25] or microbial clogging of wells [26] in iodine-producing facilities. Further studies are required to understand the physiological mechanisms underlying I_2_ tolerance by iodide-oxidizing bacteria.

## 4. Molecular Phylogeny and Systematics of *Iodidimonas*

The genus *Iodidimonas* was proposed in 2016 for iodide-oxidizing bacteria, which phylogenetically form a unique lineage near the genus *Rhodothalassium* [23]. *Iodidimonas murae* [23] and *Iodidimonas gelatinilytica* [27] have been described as members of the genus *Iodidimonas*. These are Gram-negative, aerobic, mesophilic, neutrophilic, moderately halophilic, and chemo-organotrophic bacteria. Cells form long rods, are non-sporulating and motile, and are catalase- and oxidase-positive. Iodide was oxidized on marine agar 2216. Fermentative growth was not observed. Acetate, d-glucose, maltose, sucrose, soluble starch, and yeast extract was found to support the growth of *Iodidimonas murae* as the electron donor. Q-10 was the major isoprenoid quinone. The major cellular fatty acids were C_18:1_*ω*7*c* and C_16:1_*ω*5*c*. The major polar lipids were phosphatidylethanolamine, phosphatidylglycerol, diphosphatidylglycerol, and an unidentified amino lipid. The G+C content of genomic DNA was 55 mol%. They belong to the family *Iodidimonadaceae*, order *Iodidimonadales*, and class *Alphaproteobacteria*.

**Figure 2:**
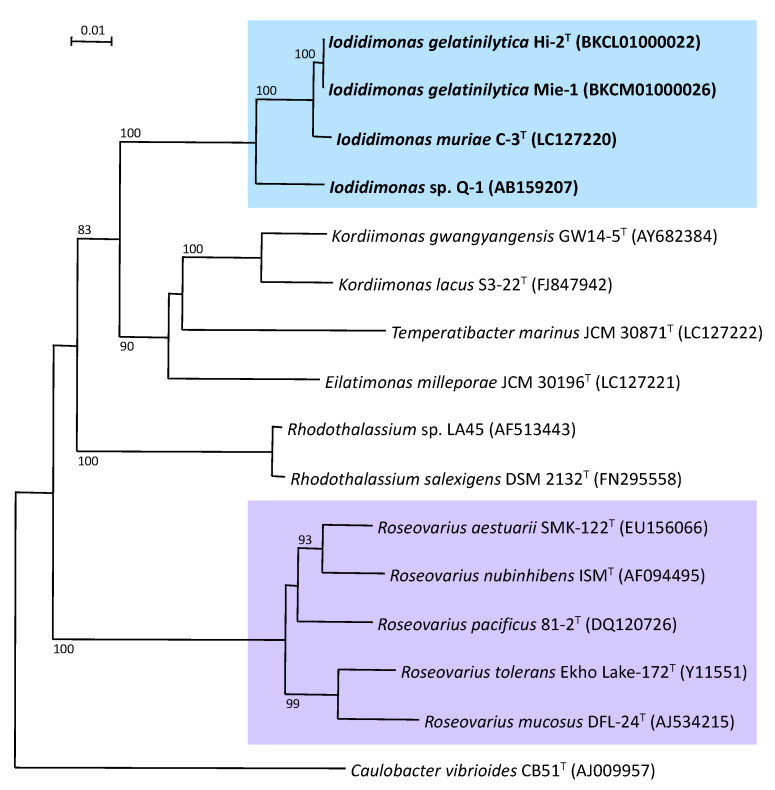
Phylogenetic tree of members of the genus *Iodidimonas* based on the 16S rRNA gene sequences. The tree was constructed using the neighbor-joining method. Numbers at nodes are bootstrap percentages derived from 1000 replications, and values of 70% or more are shown. Bar: 0.01 substitutions per nucleotide position.

The 16S rRNA gene sequence analysis was completely separated from phylogenetically-related bacteria, including the orders *Kordiimonadales* and *Rhodothalassiales* (Figure 2). Phenotypically, iodide oxidation is a unique characteristic of members of the genus *Iodidimonas*, whereas iodide does not support growth as the sole electron donor for chemolithoautotrophic growth. Furthermore, the genus *Iodidimonas* differed in the composition of major cellular fatty acids, such as C_16:0_, C_16:1_*ω5c*, C_16:1_*ω*7*c*, C_18:1_*ω*7*c*, C_18:1_*ω*7*c* 11-methyl, iso-C_15:0_, iso-C_17:0_, and iso-C_17:1_*ω*9*c*, from related bacteria including the genera *Kordiimonas*, *Eilatimonas*, *Temperatibacter*, and *Rhodothalassium*.

*Iodidimonas muriae* and *I. gelatinilytica* are distinguished by the hydrolysis of aesculin and gelatin. The former hydrolyzes aesculin but does not liquefy gelatin, and the latter liquefies gelatin but does not hydrolyze aesculin (Table 1). Furthermore, cellular fatty acid profiling demonstrated that the ratios of C_16:1_*ω*5*c* and the C_17:1_*ω*6*c* of *I. muriae* strain C-3^T^ were higher than those of *I. gelatinilytica* strains Hi-2^T^ and Mie-1. The ratios of 11-methyl C_18:1_*ω*7*c* and C_18:1_ 2-OH, and summed feature 8 were lower than those of *I. gelatinilytica*. During *in-silico* DNA–DNA hybridization, the average nucleotide identity (ANI) between *I. muriae* strain C-3^T^ and *I. gelatinilytica* strains Hi-2^T^ and Mie-1 was slightly higher than the threshold of 95% [28]. However, the value of the digital DNA–DNA hybridization (dDDH) between *I. muriae* C-3^T^ and *I. gelatinilytica* strains Hi-2^T^ and Mie-1 was lower than the threshold of 70% used for prokaryotic species delineation [29].

## 5. Biochemistry of Iodide Oxidation by *Iodidimonas*

**Figure 3:**
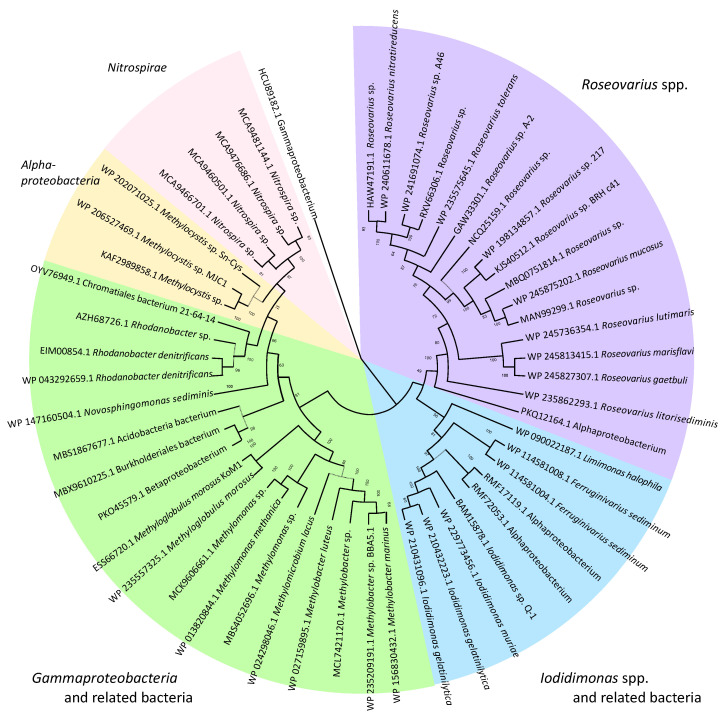
Neighbor-joining phylogenetic tree of IoxA and related proteins.

**Figure 4:**
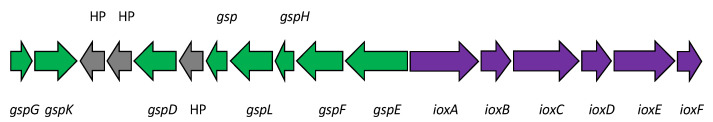
The putative *iox* gene cluster (purple) found in the draft genomes of *Iodidimonas* strains. IoxA is a multicopper oxidase and IoxB, IoxD, and IoxF are all homologs of the SCO1/SenC family protein. The functions of IoxC and IoxE are still unclear. Genes involved in the general secretion pathway (*gsp* genes, green) are present in the flanking region of the *iox* gene cluster. HP, hypothetical proteins (gray).

The iodide-oxidizing enzyme (IOX) is an extracellular protein that requires molecular oxygen, but not hydrogen peroxide, as the electron acceptor [21]. Suzuki et al. [30] purified IOX from the culture supernatant of *Iodidimonas* sp. strain Q-1. IOX showing significant activities toward iodide and various phenolic compounds such as ABTS [2,2′ azinobis (3-ethylbenzthiazolinesulfonic acid)], syringaldazine [*N,N*’-bis(3,5-dimethoxy-4-hydroxybenzylidene hydrazine)], 2,6-dimethoxy phenol, *p*-phenylenediamine, hydroquinone, and *o*-dianisidine. IOX contains copper and zinc atoms as prosthetic groups and exhibited UV/VIS absorption peaks at 320 and 590 nm. A comparison of several internal amino acid sequences obtained from trypsin-digested IOX with a draft genome sequence of strain Q-1 [31] revealed that the proteins encoded by *ioxA* and *ioxC* with predicted molecular masses of 62 and 71 kDa, respectively, were involved in iodide oxidation. Among these proteins, IoxA is closely related to a family of multicopper oxidases and includes four copper-binding regions that are highly conserved among various multicopper oxidases. However, the phylogenetic analysis demonstrated that IoxA is distantly related to previously known bacterial multicopper oxidases such as CotA, CueO, CumA, and CopA [30]. As shown in Figure 3, IoxA and related proteins were widely distributed in both the *Iodidimonas* and *Roseovarius* species, as well as in Gammaproteobacteria and Nitrospirae. Analysis of the flanking region of *ioxA* revealed that six possible ORFs (*ioxA*, *B*, *C*, *D*, *E*, and *F*) were present in the same orientation and with a relatively close sequential arrangement (Figure 4). Although the possible function of IoxC is still unclear, the proteins encoded by *ioxB*, *ioxD*, and *ioxF* demonstrated homology with SCO1/SenC family proteins, which are known to bind copper and are involved in the assembly of the cytochrome *c* oxidase complex in yeast [32]. IOX of the *Roseovarius* sp. strain A-2 was also characterized [33]. It presented characteristics similar to the IOX of strain Q-1. Both IoxA and IoxC were detected in the enzyme. These results suggest that IOX is a multicopper oxidase and that it may occur as a multimeric complex in which at least two proteins (IoxA and IoxC) are associated. As multicopper oxidases couple the oxidation of substrates with a four-electron reduction in molecular oxygen to form water [34], the reaction catalyzed by IOX is considered to be the same as Equation (1).

## 6. Genome Analysis of *Iodidimonas*

Thus far, four draft genome sequences of *Iodidimonas* strains have been published (Table 2). Their average size was 2.94 Mb with an average G+C content of 55.6%. The average number of protein-coding genes (CDSs) was 2715. All the genomes contained a putative *iox* gene cluster (*ioxA*, *B*, *C*, *D*, *E*, and *F*), suggesting that the presence of these genes is a typical characteristic of the *Iodidimonas* genomes. Interestingly, genes involved in the general secretion pathway (*gsp* genes) were present in the flanking region of the *iox* gene cluster (Figure 4), although it is unclear whether these *gsp* genes are required for IOX secretion into the extracellular environment. Various proteins involved in aerobic metabolism were identified, including NADH dehydrogenase, succinate dehydrogenase, cytochrome *c* oxidase, and superoxide dismutase. Although all *Iodidimonas* strains were catalase-positive, only strain Q-1 harbored a typical catalase-peroxidase gene. Strain Q-1 genome also contains one continuous 45,135-bp-long photosynthetic gene cluster [31], whereas the genomes of other *Iodidimonas* strains do not. This gene cluster may be acquired by a horizontal gene transfer event from closely related photosynthetic bacteria such as *Rhodothalassium salexigens*. Since the strain Q-1 genome did not contain autotrophic CO_2_ fixation pathway genes such as ribulose-1, 5-bisphosphate carboxylase/oxygenase and components of the reductive tricarboxylic acid (TCA) cycle, it may be an aerobic anoxygenic phototrophic bacterium.

## 7. Habitat and Distribution of *Iodidimonas* in Nature

As mentioned previously, *Iodidimonas* strains were first isolated from brine water associated with water-dissolved natural gas in Japan [21]. Brine was pumped up by wells from formations hundreds of meters below the surface and separated from the methane at the surface iodine production facilities. *Iodidimonas* strains are frequently isolated from brine in contact with an aerobic environment. However, *Iodidimonas* strains could not be isolated from brine freshly collected from wells [Amachi, S., unpublished results]. Other researchers have also detected or isolated *Iodidimonas* strains from aerobic brine and proposed that they are involved in the microbial deterioration of iodine production facilities [25,26].

In oil and gas production, large volumes of waste brine are generated, known as produced water (PW) or flowback. Like natural gas brine, PW often contains a substantial amount of iodide [35,36] and is thus an ideal habitat for the *Iodidimonas* species. Mohan et al. [35] first found that *Iodidimonas* and *Roseovarius* species were predominant in certain PW impoundments. In some cases, their relative abundance accounted for 65–74% of the total bacteria in the PW. A similar observation was reported by Almaraz et al. [36], where the relative abundances of the genera *Roseovarius* and *Iodidimonas* in PW treated with biologically active filtration were 52% and 6%, respectively. Van Houghton et al. [37] also reported that the relative abundance of the genera *Roseovarius* and *Iodidimonas* was more than 50% in PW treated in a membrane bioreactor. *Iodidimonas* species were also found to be the predominant bacteria in landfill leachate treated in a semi-aerobic aged refuse biofilter [38]. Furthermore, the relative abundances of *Iodidimonas* and uncultured *Iodidimonadaceae*-related bacteria were 2% and 7%, respectively, in the Karmadon springs, a geothermal site in North Caucasus [39]. These results suggest that *Iodidimonas* species are widely distributed and predominate in aerobic, saline, and iodide-rich environments. One exception is the Karmadon springs, which are freshwater environments. Thus, it might be possible that *Iodidimonas* species are more cosmopolitan bacteria than previously thought.

## 8. Potential Application of *Iodidimonas*

Iodine has strong oxidizing power and is effective as a disinfectant against bacteria, filamentous fungi, yeasts, viruses, and certain bacterial spores [40,41]. Its antimicrobial activity is rapid, less corrosive to metals, and effective even at low temperatures. Iodine has stronger bactericidal power than benzalkonium chloride and has fewer disadvantages than chlorine-based disinfectants. Based on these properties, IOX of *Iodidimonas* sp. strain Q-1 can be applied as a novel enzyme-based antimicrobial system when used with iodide. Certain peroxidases oxidize iodide to iodine; however, they require toxic hydrogen peroxide [42,43]. In contrast, the IOX/iodide system uses oxygen as an electron acceptor, making it a simpler and safer enzyme preparation method. Yuliana et al. [44] examined the antimicrobial spectrum of the IOX/iodide system and found that it could completely kill various Gram-negative bacteria, *Staphylococcus aureus*, and *Saccharomyces cerevisiae* within 5 min. The system killed *Aspergillus niger* within 10 min. The sporicidal activity of the IOX/iodide system was compared to that of povidone-iodine (PVP-I), an iodophore. PVP-I was most effective at a concentration of 0.1% [41]. However, the IOX/iodide system presented superior sporicidal activity against *Bacillus cereus*, *Bacillus subtilis*, and *Geobacillus stearothermophilus* spores compared to 0.1% PVP-I. For example, the percentage of *G. stearothermophilus* spores killed by 300 mU mL^−1^ of IOX at 120 min was 99.9%, whereas the percentage killed by 0.1% PVP-I was only 65%. This was likely because the IOX/iodide system produced a much higher level (41.2 mg L^−1^) of free iodine (I_2_) than 0.1% PVP-I (25.5 mg L^−1^). Free iodine can iodinate tyrosine residues and oxidize cysteine and histidine residues of microbial proteins. Further studies are needed to understand why IOX is not denatured or inactivated by the high concentrations of free iodine produced.

**Figure 5:**
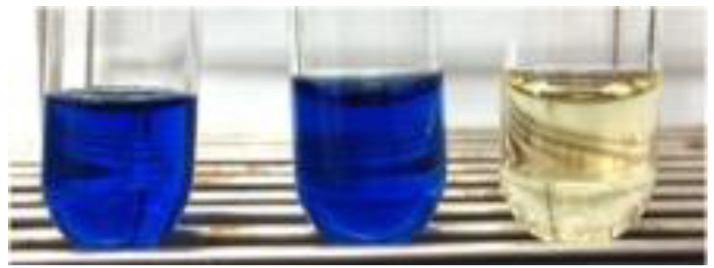
Decolorization of indigo carmine by the IOX/iodide system. The reaction mixture contained 10 mU mL^−1^ of IOX, 0.3 mM indigo carmine, 0.1 mM iodide, and 20 mM sodium acetate buffer (pH 5.5). The reaction time was 5 h. The left and middle tubes are negative controls in which IOX and iodide were omitted from the reaction mixture, respectively.

Laccase, a type of multicopper oxidase, has a decolorizing ability for a wide variety of synthetic dyes [45,46]. Thus far, many fungal laccases have been extensively studied, but their narrow range of pH and low stability against temperature and salt must be overcome in advance of industrial and biotechnological applications. Bacterial multicopper oxidases have recently attracted attention because of their excellent stability, tolerance, and ease of overexpression using genetic engineering techniques. Redox mediators such as ABTS and HOBt (1-hydroxybenzotriazole) sometimes decrease the substrate specificity of laccases. Furthermore, their high cost, toxicity, and potential impact on the natural environment remain controversial. Taguchi et al. [47] examined the potential capacity of IOX for the decolorization of anionic dyes such as orange G, indigo carmine, amido black, and remazol brilliant blue R. Interestingly, IOX efficiently decolorized all these dyes only in the presence of iodide, while no decolorization was observed in the absence of iodide (Figure 5). These results strongly suggest that iodide functions as a redox mediator in this decolorization system. The IOX/iodide decolorization system showed more alkaline pH optima and a stronger salt tolerance than laccases of fungal origin. Ebihara et al. [48] found that the IOX/iodide system was also effective for the decolorization of cationic dyes such as malachite green, crystal violet, and methylene blue under alkaline conditions. The decolorization products of malachite green were less toxic against *Escherichia coli* than malachite green. These results suggest that the IOX/iodide system is efficient for the decolorization and detoxification of various synthetic dyes. Furthermore, the IOX/iodide system may be more advantageous than the classical fungal laccase-mediator system because iodide is naturally occurring, non-toxic, and cheaper than common synthetic mediators.

Finally, *Iodidimonas* strains or their enzymes (IOXs) could be used for the bioleaching of gold from low-grade ores. Traditionally, cyanide leaching has been a major hydrometallurgical process for the recovery of gold from ores because of its high efficiency. However, cyanide is extremely toxic to humans and animals and has a serious environmental impact. Iodine gold leaching has recently attracted attention [49]. In the presence of excess iodide, I_2_ forms a triiodide ion (I_3_^−^).

I_2_ + I^−^ → I_3_^−^

Gold can be oxidized and dissolved in the mixture of iodide and triiodide to form gold (I) diiodide and/or gold (III) tetraiodide [50,51].

2Au + I_3_^−^ + I^−^ → 2[AuI_2_]^−^

2Au + 3I_3_^−^ → 2[AuI_4_]^−^ + I^−^

Khaing et al. [50] isolated iodide-oxidizing *Roseovarius* strains from brine and incubated them in a liquid medium containing iodide and ore with a gold content of 0.26% (*w/v*). Several strains showed 100% leaching of the ore within 30 d via I_3_^−^ formed from iodide. Kudpeng et al. [51] used *Roseovarius tolerans* DSM11457 and *Roseovarius mucosus* DSM17069 for bioleaching of sulfidic gold ore concentrate and electronic waste (e-waste) containing 45 and 1030 ppm gold, respectively. Although both of these bacteria were effective for leaching from the ore concentrate, the yields remained low (0.9 to 1.6%) for e-waste. Thus far, only *Roseovarius* strains were examined for the bioleaching of gold ore, but *Iodidimonas* strains and their IOXs could be tested as alternative options.

## 9. Future Aspects

Recently, Esposti et al. [52] predicted the Alphaproteobacterial origin of mitochondria (protomitochondria) by means of novel multiple phylogenomic and molecular approaches. They carefully determined: (1) M16B (a zinc peptidase)-ISP (Rieske iron sulfur protein) synteny in complex III (cytochrome *bc*_1_ complex) genes; (2) the distribution of 18 traits of mitochondrial aerobic metabolism such as complex IV (COX) genes and SCO gene; (3) the distribution of genes for ceramide and kynurenin biosynthesis; (4) the distribution of anaerobic traits such as 2-oxoacid ferredoxin oxidoreductase and *de novo* synthesis of rhodoquinone (RQ); and (5) the number of insertions or deletions (INDELs) in the catalytic subunits of complex III and IV as well as in two subunits of complex I (Nuo). Interestingly, these multiple determinations revealed that *Iodidimonas* species are superior to other possible Alphaproteobacterial candidates since they are repeatedly selected by completely different approaches. Esposti et al. [52] thus hypothesized that *Iodidimonas* species may be a descendant of the ancestral bacteria that originated protomitochondria. Although we have no available information on the putative contribution of iodine or iodide oxidation to eukaryogenesis, it is of great interest to conceive the ecology of a mitochondrial ancestor that might oxidize iodide in proteorozoic oceans or hydrothermal environments, both of which could be enriched with iodine [53].

## Figures and Tables

**Table 1:** Differential characteristics of *Iodidimonas muriae* and *Iodidimonas gelatinilytica*; Strains: 1, *Iodidimonas muriae*; 2, *Iodidimonas gelatinilytica*. +, Positive; w, weakly positive; −, negative.

Characteristic	1	2
Hydrolysis of aesculin	+	−
Hydrolysis of gelatin	−	+
API 20E		
L-Arabinose	−	+
D-Maltose	+	w/−
Cellular fatty acid composition (%)		
C_16:1_*ω*5*c*	17.2	11.5–13.4
C_17:1_*ω*6*c*	8.4	4.0–4.2
11-methyl C_18:1_*ω*7*c*	3.0	4.2–7.5
C_18:1_ 2-OH	8.4	10.8–14.1
Summed feature 8 ^a^	38.0	42.0–49.1

^a^ Summed feature represents a mixture of fatty acids that cannot be separated by the MIDI system. Summed feature 3 contained C_16:1_*ω*7*c* and/or C_16:1_*ω*6*c*. Summed feature 8 contained C_18:1_*ω*7*c*.

**Table 2:** Genome assembly and annotation data of *Iodidimonas* strains.

	Strain	Assembly	Size (Mb)	GC (%)	CDS	Contigs
*Iodidimonas muriae*	C-3^T^	GCA_014647255.1	3.01	55.6	2718	26
*Iodidimonas gelatinilytica*	Hi-2^T^	GCA_008579125.1	2.85	55.4	2709	30
*Iodidimonas gelatinilytica*	Mie-1	GCA_008579145.1	2.80	55.3	2644	47
*Iodidimonas* sp.	Q-1	GCA_000710935.1	3.09	56.1	2788	109
